# Methylation-regulated miR-374a-5p and miR-374b-5p suppress glycolysis and malignant progression of head and neck squamous cell carcinoma by targeting DEPDC1

**DOI:** 10.3389/fonc.2026.1816226

**Published:** 2026-05-15

**Authors:** Linshi Zhang, Yan Lu, Chunhong Qin, Hao Gu, Jiangfeng Du, Huina Guo, Ping Wang, Yongyan Wu, Wei Gao

**Affiliations:** 1 Department of Thyroid Surgery, The Second Affiliated Hospital of Zhejiang University School of Medicine, Hangzhou, Zhejiang, China; 2 Department of Otolaryngology Head & Neck Surgery, The First Hospital, Jinzhou Medical University, Jinzhou, Liaoning, China; 3 Shanxi Key Laboratory of Otorhinolaryngology Head and Neck Cancer, Department of Otolaryngology Head & Neck Surgery, First Hospital of Shanxi Medical University, Taiyuan, Shanxi, China; 4 Life Sciences and Medicine & Healthcare Research Institute, Northwest A&F University Shenzhen Research Institute, Shenzhen, Guangdong, China; 5 Shenzhen Institute of Otolaryngology & Key Laboratory of Otolaryngology, Longgang Otolaryngology Hospital, Shenzhen, Guangdong, China

**Keywords:** biomarker, DNA methylation, glycolysis, head and neck squamous cell carcinoma, microRNA, migration and invasion

## Abstract

Head and neck squamous cell carcinoma (HNSCC) ranks sixth in incidence among all cancers and is associated with poor prognosis. The aggressive nature of the disease, characterized by a high risk of invasion and lymph node metastasis, contributes significantly to its unfavorable prognosis. Although both microRNAs (miRNAs) and glycolysis are known to play important roles in tumor progression, the regulatory effects and mechanisms of miRNAs on glycolysis in HNSCC remain largely unclear. In this study, we analyzed the expression levels of miR-374a-5p and miR-374b-5p in HNSCC using data from TCGA and GEO databases. Functional assays included CCK-8 for cell proliferation, Transwell for migration and invasion, and Seahorse extracellular flux analysis for glycolytic capacity. *In vivo* tumorigenicity was evaluated using a nude mouse xenograft model. Mechanistic investigations were performed through qPCR, western blotting, and dual-luciferase reporter assays. Additionally, the mechanisms underlying the downregulation of miR-374a-5p and miR-374b-5p in HNSCC were investigated. Our results showed that miR-374a-5p and miR-374b-5p levels were significantly downregulated in HNSCC tissues compared with normal controls. Overexpression of either miRNA inhibited proliferation, migration, invasion, and tumor growth *in vivo*, and reduced cellular glycolytic activity. Mechanistically, miR-374a-5p and miR-374b-5p were found to bind to the 3′UTR of DEPDC1, which is upregulated in HNSCC, thereby suppressing its expression. Further experiments confirmed that DEPDC1 promotes proliferation, migration, invasion, and glycolysis in HNSCC cells, whereas miR-374a-5p and miR-374b-5p exert their inhibitory effects by downregulating DEPDC1. Furthermore, promoter methylation was identified as a key mechanism suppressing the expression of miR-374a-5p and miR-374b-5p in HNSCC cells. These findings provide novel insights and highlight potential molecular targets for HNSCC treatment through the modulation of glycolytic metabolism.

## Introduction

Over the past decade, the global incidence of head and neck squamous cell carcinoma (HNSCC) has risen significantly, accounting for 5.7% of cancer-related deaths worldwide (1). With approximately 700,000 new cases reported annually, HNSCC is characterized by high incidence and mortality rates, as well as unfavorable prognosis. Major risk factors such as tobacco use, alcohol consumption, and human papillomavirus (HPV) infection have been established, yet the precise molecular pathogenesis remains incompletely understood (2). Notably, about 64% of HNSCC patients are diagnosed at advanced stages (3). Treatment strategies-including surgery, chemotherapy, radiotherapy, combination therapy, targeted therapy, and immunotherapy-are tailored according to tumor site, stage, and patient age. However, the overall 5-year survival rate remains around 50%, and surgical intervention often leads to impaired swallowing and speech function (4). Radiotherapy is associated with side effects such as xerostomia and dysphagia, while chemoresistance contributes to postoperative recurrence and lymph node metastasis (5). These challenges underscore the need for a deeper understanding of the molecular mechanisms driving HNSCC initiation and progression, which may ultimately lead to improved therapeutic strategies and better patient outcomes.

MicroRNAs (miRNAs) are small, single-stranded, non-coding RNAs approximately 19–25 nucleotides in length, representing the smallest members of the non-coding RNA family with high evolutionary conservation. miRNAs play a key role in regulating gene expression in eukaryotic cells and are estimated to control over 60% of human genes involved in diverse biological processes such as cell cycle, differentiation, development, and metabolism (6). Moreover, miRNAs are implicated in tumor biology by modulating cancer cell growth, metabolism, invasion, metastasis, and resistance to radiotherapy and chemotherapy, often through suppression of downstream target genes (7). Human miR-374a-5p and miR-374b-5p are derived from the FTX gene transcript and share identical seed regions. Previous studies have shown that miR-374a-5p is downregulated in non-small cell lung cancer, where its overexpression inhibits cancer cell proliferation and migration (8). Similarly, upregulation of miR-374b-5p has been reported to suppress the proliferation, migration, and invasion of pancreatic cancer cells (9). Nevertheless, the expression profiles, functional roles, and regulatory mechanisms of miR-374a-5p and miR-374b-5p in HNSCC remain largely unknown.

In this study, we analyzed the expression levels of miR-374a-5p and miR-374b-5p in HNSCC tissues using transcriptomic data from The Cancer Genome Atlas (TCGA) and GEO databases. Through *in vitro* and *in vivo* experiments, we further investigated the biological functions and underlying regulatory mechanisms of these miRNAs in HNSCC. Additionally, the role of promoter methylation in modulating miR-374a-5p and miR-374b-5p expression was examined. Our findings provide insights that may contribute to the identification of potential diagnostic biomarkers and therapeutic targets for HNSCC.

## Materials and methods

### Ethical statement

The protocol used to conduct animal experiments was approved by the Animal Care Commission of Experimental Animal Center of Shanxi Medical University. Great care was taken to lessen the suffering of the included animals.

### Cell culture

Both FD-LSC-1 and AMC-HN-8 cell lines are representative cell models that are commonly used in HNSCC studies (10, 11). FD-LSC-1 cells (12) were maintained in BEGM (Lonza, Walkersville, MD, USA) containing FBS (10%) (BOVOGEN). The AMC-HN-8 cells were purchased from Shanghai Huzhen Co. (Shanghai, China) were maintained in MEM containing 10% FBS. Human oral keratinocytes (HOK) were purchased from ScienCell Research Laboratories (Carlsbad, CA) were cultured in DMEM with 10% FBS. Cell lines were authenticated by short tandem repeat analysis and tested for mycoplasma contamination using TransDetect PCR Mycoplasma Detection Kit (TransGen Biotech, Beijing, China).

### Plasmid construction and transfection

To construct the DEPDC1 3’ UTR dual−luciferase reporter plasmid, the wild−type DEPDC1 3’ UTR sequence was amplified by PCR using FD−LSC−1 cDNA as a template. Mutant versions with disrupted miR−374a−5p or miR−374b−5p binding sites were generated by overlap−extension PCR. The resulting fragments were subsequently cloned into the psiCHECK−2 vector. For the DEPDC1 overexpression plasmid, the full−length DEPDC1 coding sequence was inserted into the pcDNA3.1(+) vector. Plasmid transfection was performed using Lipofectamine 3000 reagent (ThermoFisher Scientific) in accordance with the manufacturer’s instructions.

### miRNA mimics, miRNA inhibitor, and siRNAs transfection

Negative control miRNA mimics (NC mimics), miR−374a−5p mimics, miR−374b−5p mimics, negative control miRNA inhibitor (NC inhibitor), miR−374a−5p inhibitor, miR−374b−5p inhibitor, DEPDC1−targeting siRNA (si−DEPDC1), and negative control siRNAs (si−NC) were purchased from GenePharma (Shanghai, China). Transfection of miRNA mimics, miRNA inhibitors, or siRNAs was performed using Lipofectamine 3000 reagent (ThermoFisher Scientific) according to the manufacturer’s protocol. Sequences of si-DEPDC1: si-DEPDC1-1: 5’-CCAGGAAGAUGUUGAAGAAUU-3’; 5’-UUCUUCAACAUCUUCCUGGUU-3’; si-DEPDC1-2: 5’-GGUACGAGGUCACUGAUGAUU-3’; 5’- UCAUCAGUGACCUCGUACCUU-3’.

### Gene expression analysis

Total RNA was extracted from cells using TRIzol reagent (Invitrogen, USA). For mRNA and miRNA detection, RNA was respectively reverse-transcribed into cDNA using a cDNA synthesis kit (Vazyme) and the miScript II RT Kit (Qiagen, Germany). Quantitative real-time PCR (qPCR) was performed on a StepOnePlus system (Thermo Fisher Scientific, Indianapolis, IN) with TransStart Tip Green qPCR SuperMix (Transgen Biotech) according to the manufacturer’s instructions. The expression levels of target genes were normalized to those of the internal controls Actin (for mRNA) and U6 (for miRNA). The sequences of the primers are as follows: DEPDC1: 5’-CTCCCTGAACCTCTACTTACT-3’; 5’-CTCATTCGGGAAATCATAC-3’. U6: 5’-TCGCTTCGGCAGCACATAT-3’; 5’-ATTTGCGTGTCATCCTTGC-3’. Actin: 5’-TCCCTGGAGAAGAGCTACGA-3’; 5’-AGCACTGTGTTGGCGTACAG-3’. miR-374a-5p Forward: TTATAATACAACCTGATAAGTG. miR-374b-5p Forward: ATATAATACAACCTGCTAAGTG. miRNA Reverse: CTCAACTGGTGTCGTGGA.

### Dual-luciferase reporter assay

Wild-type or mutant DEPDC1 reporter plasmids were co-transfected with NC mimics, miR-374a-5p mimics, or miR-374b-5p mimics for 48 hours. Luciferase activity was then measured using the Dual-Luciferase Reporter Assay System (Promega, Madison, WI) according to the manufacturer’s protocol.

### Western blotting

Total protein was extracted using RIPA buffer supplemented with a protease inhibitor cocktail (ThermoFisher Scientific). Equal amounts of protein were separated by SDS-PAGE and transferred onto PVDF membranes (Millipore, Bedford, MA). After blocking with 5% non-fat milk, the membranes were incubated overnight at 4 °C with primary antibodies, followed by three washes with TBST. Subsequently, the membranes were incubated with secondary antibodies at room temperature for 2 hours and washed three times with TBST. Protein bands were visualized using an ECL reagent (ZETALIFE Inc.). The primary antibody against DEPDC1 was obtained from BBI Life Sciences (Cat# D222229, 1:1000). Primary antibodies against GAPDH (Cat# 60004-1-Ig, 1:100,000), E-cadherin (Cat# 20874-1-AP, 1:20,000), Slug (Cat# 12129-1-AP, 1:5000), and Vimentin (Cat# 10366-1-AP, 1:10,000) were purchased from Proteintech.

### CCK8 assay

For the CCK−8 assay, cells (1×10³ cells/well) were seeded in 96−well plates. Cell proliferation was assessed at the indicated time points using a CCK8 kit (Yeasen Biotech, Shanghai, China) according to the manufacturer’s instructions.

### Transwell migration and invasion assay

Cells were suspended in serum−free medium. For invasion assays, Transwell chambers were pre−coated with Matrigel (BD Biosciences, San Jose, CA). A total of 100 µL of serum−free medium containing 1×10^5^ cells/well (invasion) or 4×10^4^ cells/well (migration) was seeded into the upper chamber, while 600 µL of medium supplemented with 20% FBS was added to the lower chamber. After incubation for 48 h (migration) or 72 h (invasion), the non−invading/non−migrating cells on the upper surface were removed, and the chambers were gently rinsed with PBS. Cells on the lower membrane were fixed with paraformaldehyde for 10 min and stained with 0.1% crystal violet for 15 min. Images were captured and analyzed under an inverted microscope (Leica Microsystems Inc., Buffalo Grove, IL).

### Immunohistochemical staining

Immunohistochemical (IHC) staining was performed as previously described (13). Briefly, 3 μm paraffin−embedded sections were prepared, dewaxed, and rehydrated. Following antigen retrieval and blocking, sections were incubated with primary antibodies at 4 °C overnight. Primary antibodies used included: Ki67 (Cat# 27309−1−AP, 1:5000), E−cadherin (Cat# 20874−1−AP, 1:5000), Slug (Cat# 12129−1−AP, 1:1000), and Vimentin (Cat# 10366−1−AP, 1:3000), all purchased from Proteintech. Sections were then incubated with a secondary antibody for 0.5 h at room temperature, developed with DAB solution, and counterstained with hematoxylin. Finally, sections were dehydrated, mounted with coverslips, and examined under a microscope. Whole-slide scanning was conducted using a Panoramic Slide Scanner II (3D HISTECH, Budapest, Hungary), and whole-slide image analysis was performed with QuPath v0.7.0. For each tumor, ten representative regions of interest were examined. Positive cell detection was executed by employing the built-in positive cell detection algorithm. All analyses were evaluated independently by two board-certified pathologists, with discrepancies resolved through discussion to reach a consensus. To minimize bias, the slides were anonymized, and the pathologists were blinded to all characteristics and outcomes.

### Xenograft model studies

BALB/c nude mice (female, aged 6 weeks) were obtained from Beijing Vital River Laboratory Animal Technology Co., Ltd. (Beijing, China). FD-LSC-1 cells (5×10^6^ cells/mouse) were administered subcutaneously into the right flank of every mouse. After 10 days implantation, the xenograft models were randomly assigned to three groups: six mice received intratumoral injections of 10 nmol NC mimics with *in vivo* transfection reagent (NC mimics group), three mice were administered miR-374a-5p mimics (miR-374a-5p group), and three mice received miR-374b-5p mimics (miR-374b-5p group), respectively. Injections were performed every three days. On day 26, tumor−bearing mice were anesthetized with isoflurane and euthanized by cervical dislocation. The xenograft tumors were then dissected, weighed, and photographed.

### Bioinformatics analysis

The normalized expression data (Fragments Per Kilobase Million, FPKM) of DEPDC1 in the HNSCC cohort from The Cancer Genome Atlas (TCGA) were employed for differential expression and Kaplan−Meier survival analyses. The expression levels of miR−374a−5p, miR−374b−5p, and DEPDC1 were examined using publicly available RNA−sequencing datasets of 107 cases of laryngeal squamous cell carcinoma (LSCC) tissues and paired adjacent normal mucosa from the GEO database (accessions: GSE132222, GSE130605, GSE127165, and GSE133632), as previously described (14). Putative target genes of miR−374a−5p and miR−374b−5p were predicted using TargetScanHuman 7.1 (15). Gene Ontology (GO) and Kyoto Encyclopedia of Genes and Genomes (KEGG) pathway enrichment analyses were performed with DAVID 6.8 (https://davidbioinformatics.nih.gov/home.jsp).

### Extracellular acidification rate analysis

The extracellular acidification rate (ECAR) was measured using a Seahorse XFp analyzer (Agilent Technologies, Inc., Santa Clara, CA) according to the manufacturer’s protocol. Briefly, cells were seeded into Seahorse eight−well plates at a density of 5,000 cells per well and cultured for 12 h. ECAR was then recorded following the sequential injection of 10 mM glucose, 2 μM oligomycin, and 50 mM 2−deoxy−D−glucose (2-DG).

### Glycolysis phenotype assay

Lactate levels were quantified using a microplate−based lactate assay kit (Biosharp, China), and ATP content was measured with a microplate ATP content assay kit (Biosharp, China). Both assays were performed according to the manufacturer’s protocols.

### Next generation sequencing-based bisulfite sequencing PCR (BSP)

Gene-specific DNA methylation was examined by a next generation sequencing-based BSP according to report previously (16). Briefly, BSP primers for FTX promoter were designed using the online MethPrimer software (17) (https://www.methprimer.com/). 1 μg of genomic DNA was converted using the ZYMO EZ DNA Methylation-Gold Kit (Zymo Research, Irvine, CA) and one twentieth of the elution products were used as templates for PCR amplification with 35 cycles using KAPA HiFi HotStart Uracil+ ReadyMix PCR Kit (Kapa Biosystems, Wilmington, MA). For each sample, BSP products of multiple primers were pooled equally, 5’-phosphorylated, 3’-dA-tailed and ligated to barcoded adapter using T4 DNA ligase (NEB). Barcoded libraries from all samples were sequenced on Illumina platform.

### Statistical analysis

Statistical tests were performed with the GraphPad 8 software. Data in bar and line graphs are presented as mean ± S.D. of at least three independent experiments. Statistical comparisons were performed using unpaired t-test, paired t-test, and two-way analysis of variance (ANOVA) as indicated. Overall survival was defined as the time from the date of surgery until death due to HNSCC or the last follow-up. Survival curves were generated by Kaplan–Meier analysis and evaluated with the log-rank test. A p-value of < 0.05 was considered statistically significant.

## Results

### The expression levels of miR-374a-5p and miR-374b-5p were downregulated in HNSCC

**Figure 1:**
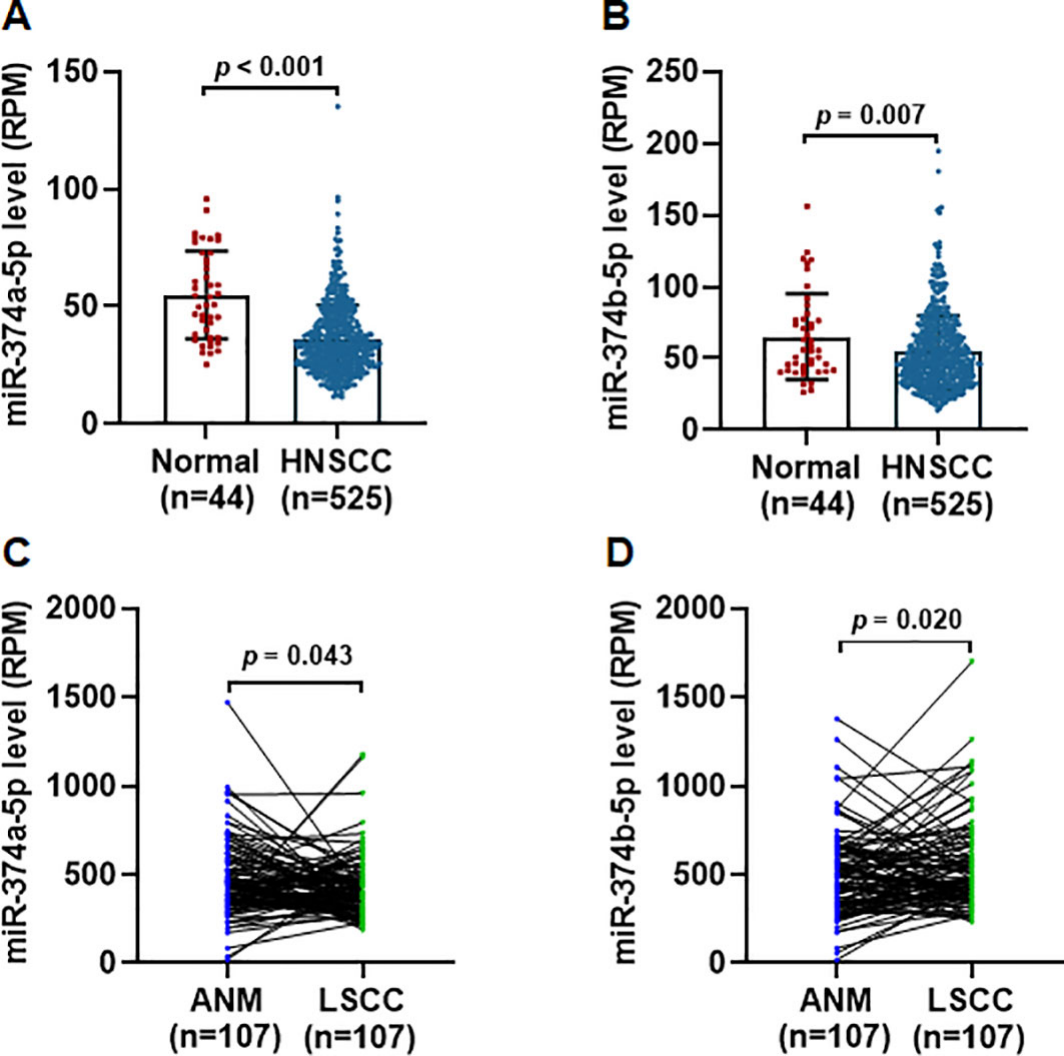
miR-374a-5p and miR-374b-5p were downregulated in HNSCC. **(A, B)** The expression levels of miR−374a−5p **(A)** and miR−374b−5p **(B)** in HNSCC tissues compared with normal tissues were analyzed using TCGA RNA-seq data. **(C, D)** The expression levels of miR−374a−5p **(C)** and miR−374b−5p **(D)** in LSCC tissues versus ANM were analyzed using RNA-seq data from GEO database. Statistical analysis was performed using unpaired t-test for panels **(A, B)**, and paired t-test for panels **(C, D)**.

We analyzed the expression of miR-374a-5p and miR-374b-5p in HNSCC and normal tissues using the TCGA database. The results showed that both miRNAs were significantly downregulated in HNSCC tissues compared with normal controls (**Figures 1A, B**). Laryngeal squamous cell carcinoma (LSCC) represents the second most common subtype of HNSCC. In this study, we also examined miR-374a-5p and miR-374b-5p expression in our previously established cohort of 107 LSCC tissues and paired adjacent normal mucosa (ANM) (14). Consistent with the TCGA HNSCC data, both miRNAs were markedly downregulated in LSCC relative to ANM (**Figures 1C, D**). These findings suggest that miR-374a-5p and miR-374b-5p may act as tumor suppressors in HNSCC.

### miR−374a−5p and miR−374b−5p inhibit the proliferation, migration, and invasion of HNSCC cells

**Figure 2:**
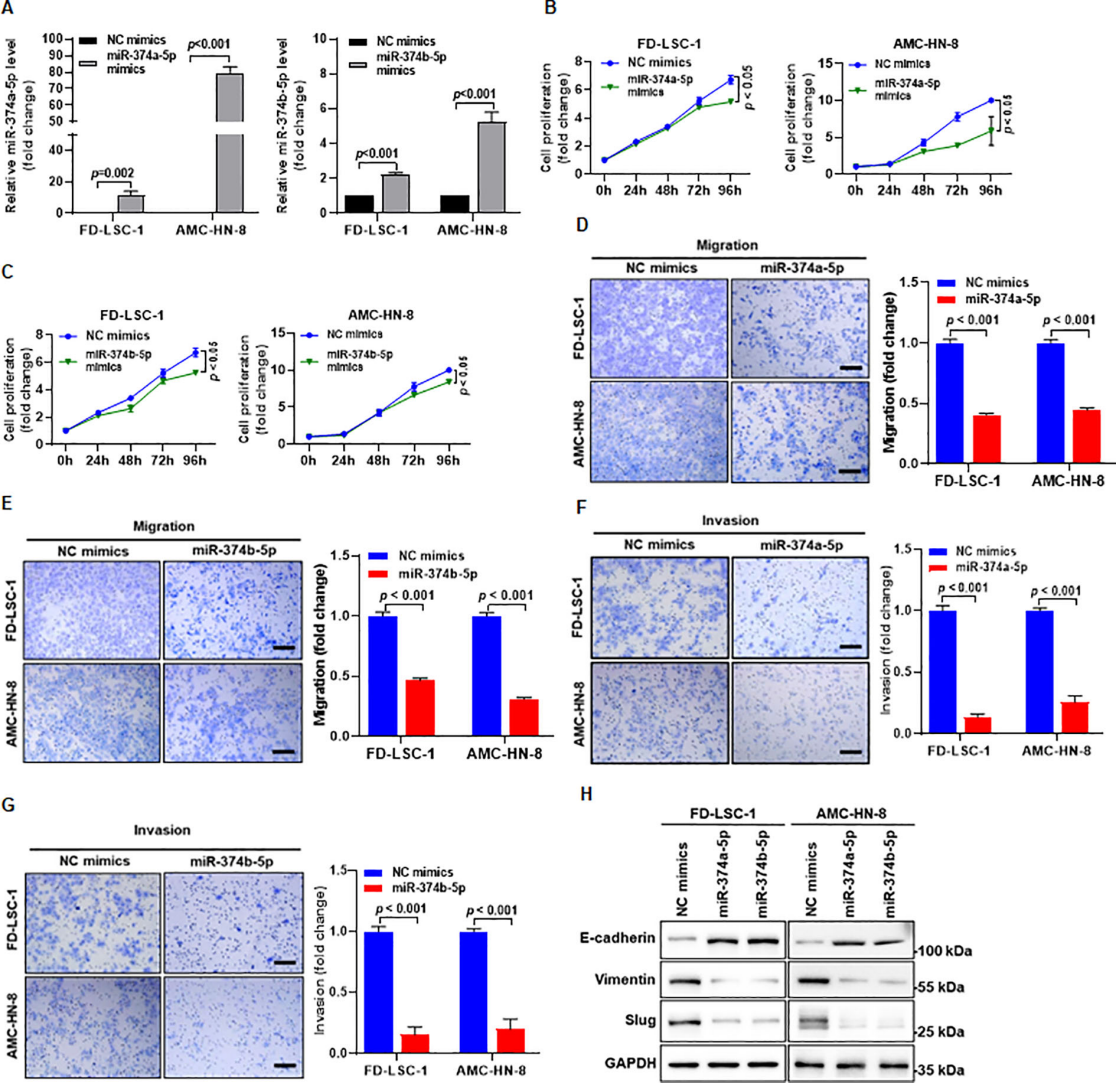
Overexpression of miR-374a-5p or miR-374b-5p suppresses the proliferation, migration, and invasion of HNSCC cells. **(A)** HNSCC cells were transfected with miR−374a−5p mimics, miR−374b−5p mimics, or negative control (NC) mimics. The expression levels of miR−374a−5p and miR−374b−5p were measured by qPCR 48 h after transfection. **(B)** Proliferation of FD−LSC−1 and AMC-HN-8 cells transfected with miR−374a−5p mimics or NC mimics was assessed by CCK−8 assay at the indicated time points. **(C)** Proliferation of FD−LSC−1 and AMC−HN−8 cells transfected with miR−374b−5p mimics or NC mimics was assessed by CCK−8 assay at the indicated time points. **(D, E)** Migration ability of FD−LSC−1 and AMC−HN−8 cells transfected with miR−374a−5p mimics **(D)**, miR−374b−5p mimics **(E)**, or NC mimics was evaluated by transwell assay. **(F, G)** Invasion ability of FD−LSC−1 and AMC−HN−8 cells transfected with miR−374a−5p mimics **(F)**, miR−374b−5p mimics **(G)**, or NC mimics was evaluated by transwell assay. **(H)** The expression levels of EMT markers E-cadherin, Vimentin, and Slug were detected by western blotting 48 h after transfection. Statistical analysis was performed using unpaired t-test for panels **(A, D, E, F, G)**, and two-way ANOVA for panels **(B, C)**. Scale bar, 100 μm. Data are mean ± SD of three separate experiments.

To investigate the functional roles of miR−374a−5p and miR−374b−5p in HNSCC, miR−374a−5p mimics and miR−374b−5p mimics were transfected into the HNSCC cell lines FD−LSC−1 and AMC−HN−8, respectively. Quantitative PCR confirmed that the expression levels of both miRNAs were significantly increased in the transfected cells (**Figure 2A**), indicating successful upregulation of miR−374a−5p and miR−374b−5p in HNSCC cells. Subsequently, we examined the phenotypic effects of miR−374a−5p and miR−374b−5p overexpression. CCK−8 assays showed that cell proliferation was significantly reduced upon transfection with miR−374a−5p mimics (**Figure 2B**) or miR−374b−5p mimics (**Figure 2C**). Transwell assays further revealed that the migration and invasion abilities of both FD−LSC−1 and AMC−HN−8 cells were markedly suppressed in groups transfected with miR−374a−5p or miR−374b−5p mimics compared with the control group (**Figures 2D–G**). Furthermore, western blot analysis showed that overexpression of miR−374a−5p or miR−374b−5p upregulated E−cadherin levels and downregulated Vimentin, and Slug in HNSCC cells (**Figure 2H**), indicating that both miRNAs inhibit epithelial−mesenchymal transition (EMT) in HNSCC cells. Compared with cells transfected with a negative control miRNA inhibitor, transfection with miR-374a-5p inhibitor or miR-374b-5p inhibitor significantly promoted the proliferation, migration, and invasion of HNSCC cells (**Supplementary Figure 1**). These results suggest that both miR-374a-5p and miR-374b-5p suppress the proliferation, migration, and invasion of HNSCC cells.

### Overexpression of miR−374a−5p or miR−374b−5p inhibits *in vivo* tumor growth of HNSCC cells

**Figure 3:**
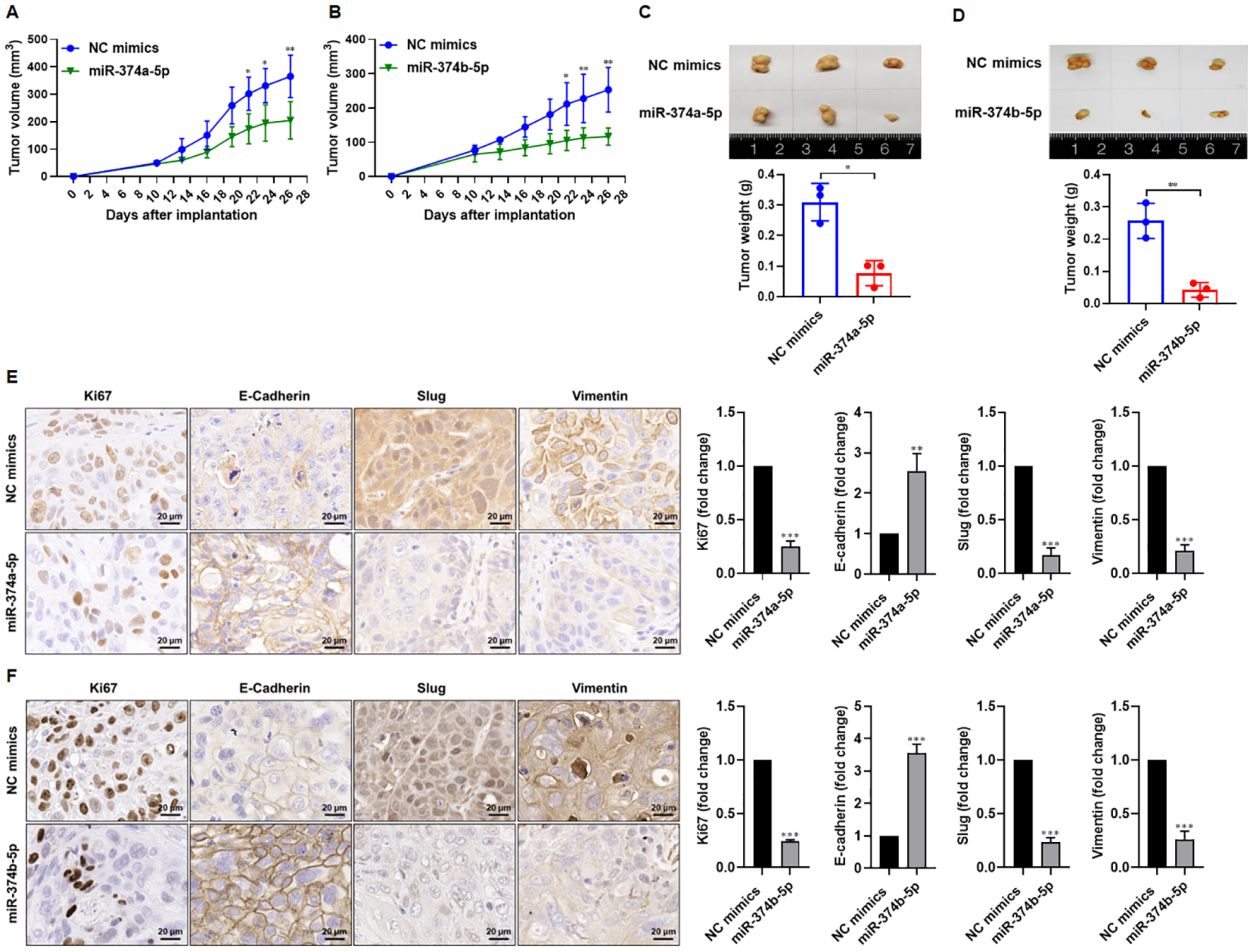
Overexpression of miR−374a−5p or miR−374b−5p suppresses tumorigenesis in HNSCC cells. A xenograft model was established by subcutaneous injection of FD−LSC−1 cells into nude mice. After tumor formation, miR−374a−5p mimics, miR−374b−5p mimics, or negative control (NC) mimics was injected intratumorally using an *in vivo* transfection reagent. Tumor growth curves of mice treated with miR−374a−5p mimics **(A)** or miR−374b−5p mimics **(B)**. Representative images and tumor weights of xenografts from mice injected with miR−374a−5p mimics **(C)** or miR−374b−5p mimics **(D)**. **(E)** Immunohistochemical (IHC) staining was performed to detect the expression of the proliferation marker Ki67 and EMT markers (E−cadherin, Slug, and Vimentin) in xenografts treated with miR−374a−5p mimics. Representative images (left) and quantified data (right) are shown. **(F)** IHC staining for Ki67, E−cadherin, Slug, and Vimentin in xenografts treated with miR−374b−5p mimics. Representative images (left) and quantified data (right) are shown. Statistical analysis was performed using two-way ANOVA for panels **(A, B)**, and unpaired t-test for panels **(C–F)**. Scale bar, 20 μm. Data are mean ± SD of three separate experiments. *p < 0.05, **p < 0.01, ***p < 0.001.

Having clarified the effects of miR-374a-5p and miR-374b-5p on HNSCC cell phenotypes *in vitro*, we established a nude mouse xenograft model to assess their impact on *in vivo* tumorigenicity. FD-LSC-1 cells were subcutaneously injected into the forelimb axilla of nude mice, and tumor growth was monitored regularly. The results demonstrated that overexpression of miR-374a-5p or miR-374b-5p significantly slowed xenograft growth compared with the control group (**Figures 3A, B**). Moreover, the weight of xenografts in the miR-374a-5p or miR-374b-5p mimic groups was markedly lower than that in the NC mimic group (**Figures 3C, D**), indicating that both miRNAs suppress tumor formation of HNSCC cells *in vivo*.

To evaluate the effect of these miRNAs on *in vivo* proliferation, we performed immunohistochemical staining for the proliferation marker Ki67 in xenograft tissues. The number of Ki67−positive cells was significantly reduced in tumors overexpressing miR−374a−5p or miR−374b−5p compared with controls (**Figures 3E, F**). Given the close association between EMT and cancer cell invasion/metastasis (18), we further examined whether miR−374a−5p and miR−374b−5p modulate EMT in HNSCC. Immunohistochemical analysis revealed that xenografts expressing miR−374a−5p or miR−374b−5p mimics showed elevated levels of the epithelial marker E−cadherin and reduced expression of the mesenchymal markers Slug and Vimentin (**Figures 3E, F**). Collectively, these findings indicate that overexpression of miR−374a−5p or miR−374b−5p inhibits malignant phenotypes of HNSCC cells *in vivo*.

### Overexpression of miR-374a-5p or miR-374b-5p reduces the glycolytic activity in HNSCC cells

**Figure 4:**
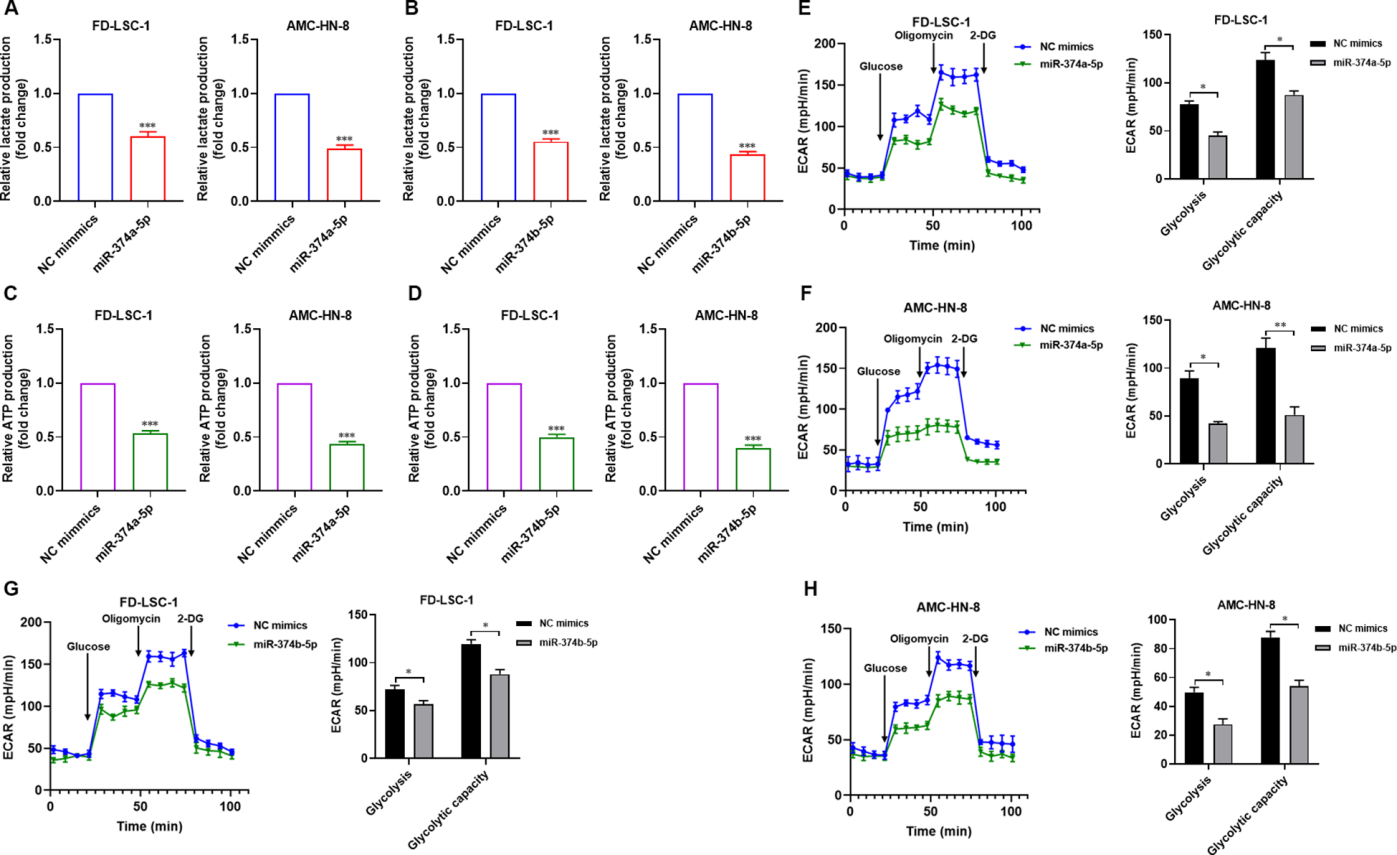
Overexpression of miR-374a-5p or miR-374b-5p suppresses glycolysis in HNSCC cells. **(A)** Measurement of relative lactate production in FD−LSC−1 and AMC-HN-8 cells transfected with miR−374a−5p mimics or NC mimics. **(B)** Measurement of relative lactate production in FD−LSC−1 and AMC-HN-8 cells transfected with miR−374b−5p mimics or NC mimics. **(C)** Measurement of relative ATP production in FD−LSC−1 and AMC-HN-8 cells transfected with miR−374a−5p mimics or NC mimics. **(D)** Measurement of relative ATP production in FD−LSC−1 and AMC-HN-8 cells transfected with miR−374b−5p mimics or NC mimics. **(E)** Measurement of ECAR in FD−LSC−1 cells transfected with miR−374a−5p mimics or NC mimics. **(F)** Measurement of ECAR in AMC-HN-8 cells transfected with miR−374a−5p mimics or NC mimics. **(G)** Measurement of ECAR in FD−LSC−1 cells transfected with miR−374b−5p mimics or NC mimics. **(H)** Measurement of ECAR in AMC-HN-8 cells transfected with miR−374b−5p mimics or NC mimics. Statistical analysis was performed using unpaired t-test for panels **(A–H)**. Data are mean ± SD of three separate experiments. *p < 0.05, **p < 0.01, ***p < 0.001.

Glycolysis serves as a preferred metabolic pathway in tumor cells, which typically rely on glycolytic metabolism for energy supply regardless of oxygen availability. Our previous study demonstrated that enhanced glycolysis promotes proliferation, invasion, migration, and chemotherapy resistance in LSCC cells (13). We therefore investigated whether miR-374a-5p and miR-374b-5p influence glycolysis in HNSCC cells. Lactate accumulation and ATP production were measured as key glycolytic readouts. Overexpression of either miR-374a-5p or miR-374b-5p in FD−LSC−1 and AMC−HN−8 cells led to pronounced suppression of glycolysis, characterized by decreased lactate production (**Figures 4A, B**) and reduced ATP levels (**Figures 4C, D**). Moreover, using a Seahorse extracellular flux analyzer, we monitored the extracellular acidification rate (ECAR) in these cells. Results showed that both FD−LSC−1 and AMC−HN−8 cells transfected with miR-374a-5p mimics exhibited lower ECAR values compared with the control group (**Figures 4E, F**). Similarly, transfection with miR-374b-5p mimics also significantly decreased ECAR in both cell lines (**Figures 4G, H**). Collectively, these findings indicate that overexpression of miR-374a-5p or miR-374b-5p inhibits glycolysis in HNSCC cells.

### DEPDC1 is a direct target gene of miR-374a-5p and miR-374b-5p in HNSCC cells

**Figure 5:**
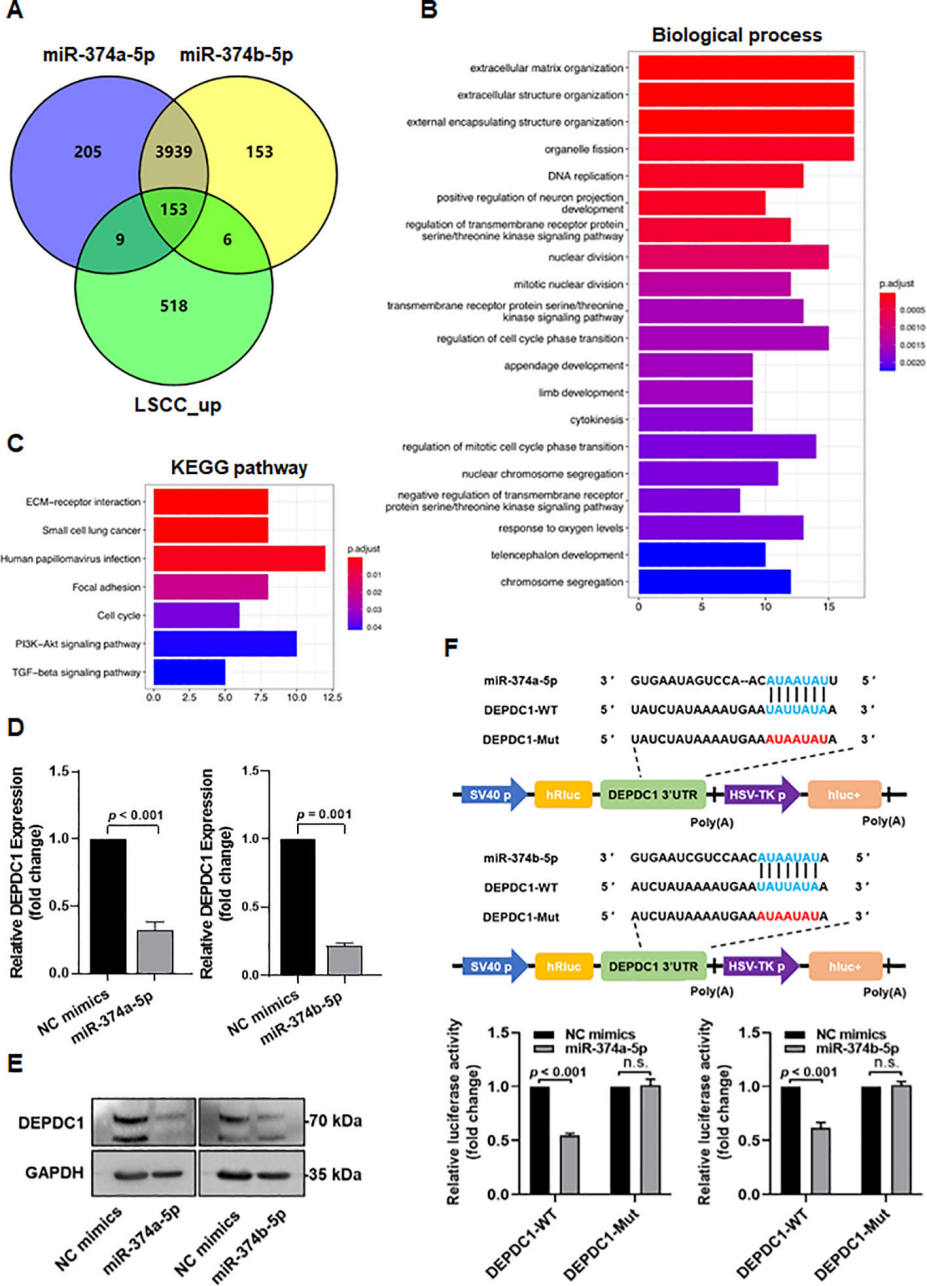
Bioinformatic analysis and experimental validation of miR-374a-5p and miR-374b-5p target genes. **(A)** Venn diagram illustrating the overlap between predicted target genes of miR−374a−5p, predicted target genes of miR−374b−5p, and upregulated genes in LSCC tissues. **(B)** Gene Ontology (GO) analysis of candidate target genes. The top 20 enriched biological processes are displayed based on gene count and p−value. **(C)** KEGG pathway enrichment analysis of candidate target genes. **(D)** Expression of DEPDC1 mRNA in xenograft tumors injected with miR−374a−5p mimics or miR−374b−5p mimics was measured by qPCR. **(E)** Expression of DEPDC1 protein in xenograft tumors injected with miR−374a−5p mimics or miR−374b−5p mimics was detected by western blotting. **(F)** Cells were co−transfected with miR−374a−5p mimics or miR−374b−5p mimics together with either wild−type (DEPDC1−WT) or mutant (DEPDC1−Mut) 3’−UTR reporter plasmids for 48 h, after which relative luciferase activity was determined. Statistical analysis was performed using unpaired t-test for panels **(D, F)**. Data are mean ± SD of three separate experiments.

To investigate the molecular mechanisms of miR-374a-5p and miR-374b-5p in HNSCC cells, we first performed bioinformatic prediction of their target genes. A total of 153 candidate target genes were identified (**Figure 5A**). Gene Ontology (GO) analysis indicated that these genes are primarily involved in biological processes including extracellular matrix organization, DNA replication, nuclear division, regulation of cell cycle phase transition, regulation of mitotic cell cycle phase transition, response to oxygen levels, and chromosome segregation (**Figure 5B**). Furthermore, KEGG pathway enrichment analysis demonstrated that the predicted targets are mainly associated with pathways such as ECM-receptor interaction, small cell lung cancer, human papillomavirus infection, focal adhesion, cell cycle, PI3K-Akt signaling, and TGF-beta signaling (**Figure 5C**).

Among the predicted target genes, DEPDC1 (DEP Domain Containing 1) has been demonstrated to be upregulated in breast cancer and prostate cancer, facilitating cell proliferation, migration, and invasion (19, 20), and participating in cell cycle regulation (21), which align with the biological processes and pathways associated with miR-374a-5p and miR-374b-5p. Therefore, we selected DEPDC1 as the candidate target gene to verify. First, qPCR was performed to measure the mRNA expression level of DEPDC1 in HNSCC xenograft tumors overexpressing miR−374a−5p or miR−374b−5p. Compared with the control group, overexpression of either miR−374a−5p or miR−374b−5p significantly reduced DEPDC1 mRNA expression (**Figure 5D**). Western blot analysis further confirmed that DEPDC1 protein levels were also decreased in xenograft tissues from the miR−374a−5p or miR−374b−5p groups (**Figure 5E**), indicating that both miRNAs negatively regulate DEPDC1 expression. To investigate the direct targeting mechanism, we constructed dual−luciferase reporter vectors carrying either the wild−type 3′UTR of DEPDC1 (DEPDC1−WT) or a mutant 3′UTR with disrupted binding sites for miR−374a−5p and miR−374b−5p (DEPDC1−Mut). Luciferase reporter assays demonstrated that overexpression of miR−374a−5p or miR−374b−5p significantly decreased the luciferase activity of DEPDC1−WT, but had no significant effect on DEPDC1−Mut activity (**Figure 5F**). These results suggest that DEPDC1 is a direct target of miR−374a−5p and miR−374b−5p, and that these miRNAs downregulate DEPDC1 expression by binding to its 3′UTR.

### DEPDC1 promotes proliferation, migration, invasion, and glycolytic activity of HNSCC cells

**Figure 6:**
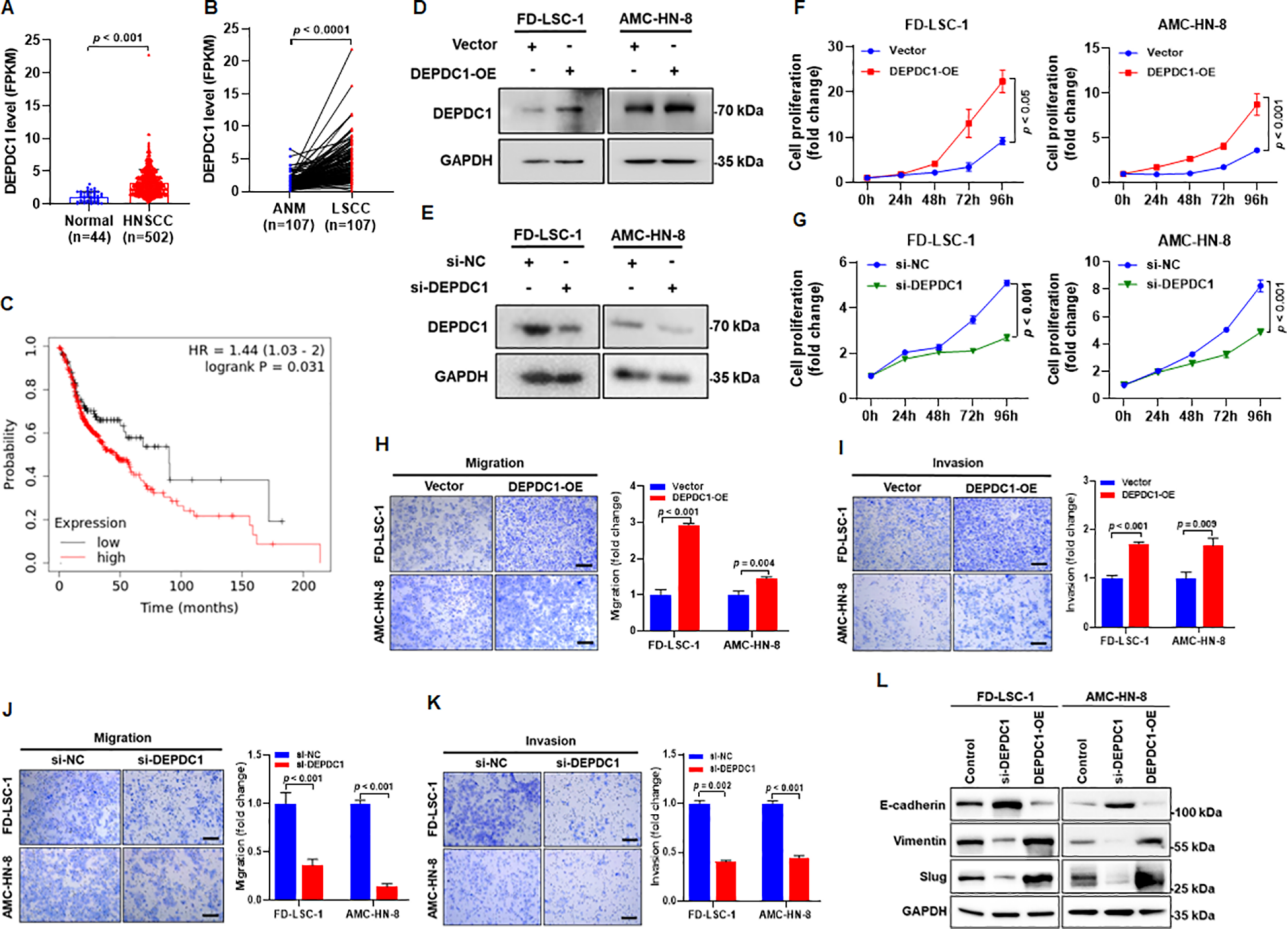
DEPDC1 is upregulated in HNSCC and is associated with malignant phenotypes of HNSCC cells. **(A)** Expression of DEPDC1 mRNA in HNSCC tissues compared with normal tissues was analyzed using RNA-seq data from the TCGA database. **(B)** Expression of DEPDC1 mRNA in LSCC tissues compared with adjacent normal mucosa was analyzed using RNA-seq data from GEO database. **(C)** Kaplan−Meier analysis showing the association between DEPDC1 expression and overall survival in HNSCC patients. Expression and clinical data were obtained from the TCGA database. The low−DEPDC1 group (n=128) and high−DEPDC1 group (n=371) are indicated. **(D)** FD−LSC−1 and AMC−HN−8 cells were transfected with a DEPDC1 overexpression plasmid (DEPDC1−OE) or an empty vector (Vector) for 48 h, followed by detection of DEPDC1 protein levels by western blotting. **(E)** FD−LSC−1 and AMC−HN−8 cells were transfected with siRNA targeting DEPDC1 (si−DEPDC1) or negative control siRNA (si−NC) for 48 h, followed by detection of DEPDC1 protein levels by western blotting. **(F)** FD−LSC−1 and AMC−HN−8 cells were transfected with DEPDC1−OE or Vector, and cell proliferation was assessed by CCK−8 assay. **(G)** FD−LSC−1 and AMC−HN−8 cells were transfected with si−DEPDC1 or si−NC, and cell proliferation was assessed by CCK−8 assay. **(H)** FD−LSC−1 and AMC−HN−8 cells were transfected with DEPDC1−OE or Vector, and cell migration was evaluated by transwell assay. **(I)** FD−LSC−1 and AMC−HN−8 cells were transfected with DEPDC1−OE or Vector, and cell invasion was evaluated by transwell assay. **(J)** FD−LSC−1 and AMC−HN−8 cells were transfected with si−DEPDC1 or si−NC, and cell migration was evaluated by transwell assay. **(K)** FD−LSC−1 and AMC−HN−8 cells were transfected with si−DEPDC1 or si−NC, and cell invasion was evaluated by transwell assay. **(L)** The expression levels of EMT markers E-cadherin, Vimentin, and Slug were detected by western blotting. Statistical analysis was performed using unpaired t-test for panels **(A, H–K)**, paired t-test for panel **(B)**, two-way ANOVA for panels **(F, G)**. Scale bar, 100 μm. Data are mean ± SD of three separate experiments.

Analysis of RNA-seq data from the TCGA HNSCC cohort revealed significantly higher DEPDC1 expression in tumor tissues compared with normal controls (**Figure 6A**). Consistent with this, RNA−seq data from a previously described cohort (14) also demonstrated elevated DEPDC1 levels in LSCC tissues relative to matched ANM (**Figure 6B**). Kaplan−Meier survival analysis further indicated that HNSCC patients with low DEPDC1 expression had significantly longer overall survival than those with high DEPDC1 expression (**Figure 6C**), suggesting a potential oncogenic role for DEPDC1 in HNSCC.

To explore the biological function of DEPDC1, HNSCC cells were transfected with either DEPDC1 overexpression plasmids or siRNA targeting DEPDC1. Western blot analysis confirmed that DEPDC1 protein levels were increased upon overexpression (**Figure 6D**). In contrast, transfection with si-DEPDC1–1 and si-DEPDC1–2 significantly reduced the expression level of DEPDC1, with si-DEPDC1–1 showing higher knockdown efficiency than si-DEPDC1-2 (**Figure 6E**, **Supplementary Figure 2**). Therefore, si-DEPDC1–1 was selected for use in subsequent experiments and is referred to as si-DEPDC1. CCK−8 assays revealed that DEPDC1 overexpression enhanced HNSCC cell proliferation (**Figure 6F**), whereas DEPDC1 knockdown suppressed it (**Figure 6G**). Transwell experiments demonstrated that DEPDC1 overexpression increased the migratory and invasive abilities of FD−LSC−1 and AMC−HN−8 cells (**Figures 6H, I**), while DEPDC1 knockdown reduced both migration (**Figure 6J**) and invasion (**Figure 6K**). Western blot analysis showed that DEPDC1 knockdown elevated the expression of the epithelial marker E−cadherin and lowered that of the mesenchymal markers Vimentin and Slug, whereas DEPDC1 overexpression produced the opposite pattern (**Figure 6L**), indicating that DEPDC1 promotes EMT in HNSCC cells. Together, these findings demonstrate that DEPDC1 enhances the proliferation, migration, and invasion of HNSCC cells.

**Figure 7:**
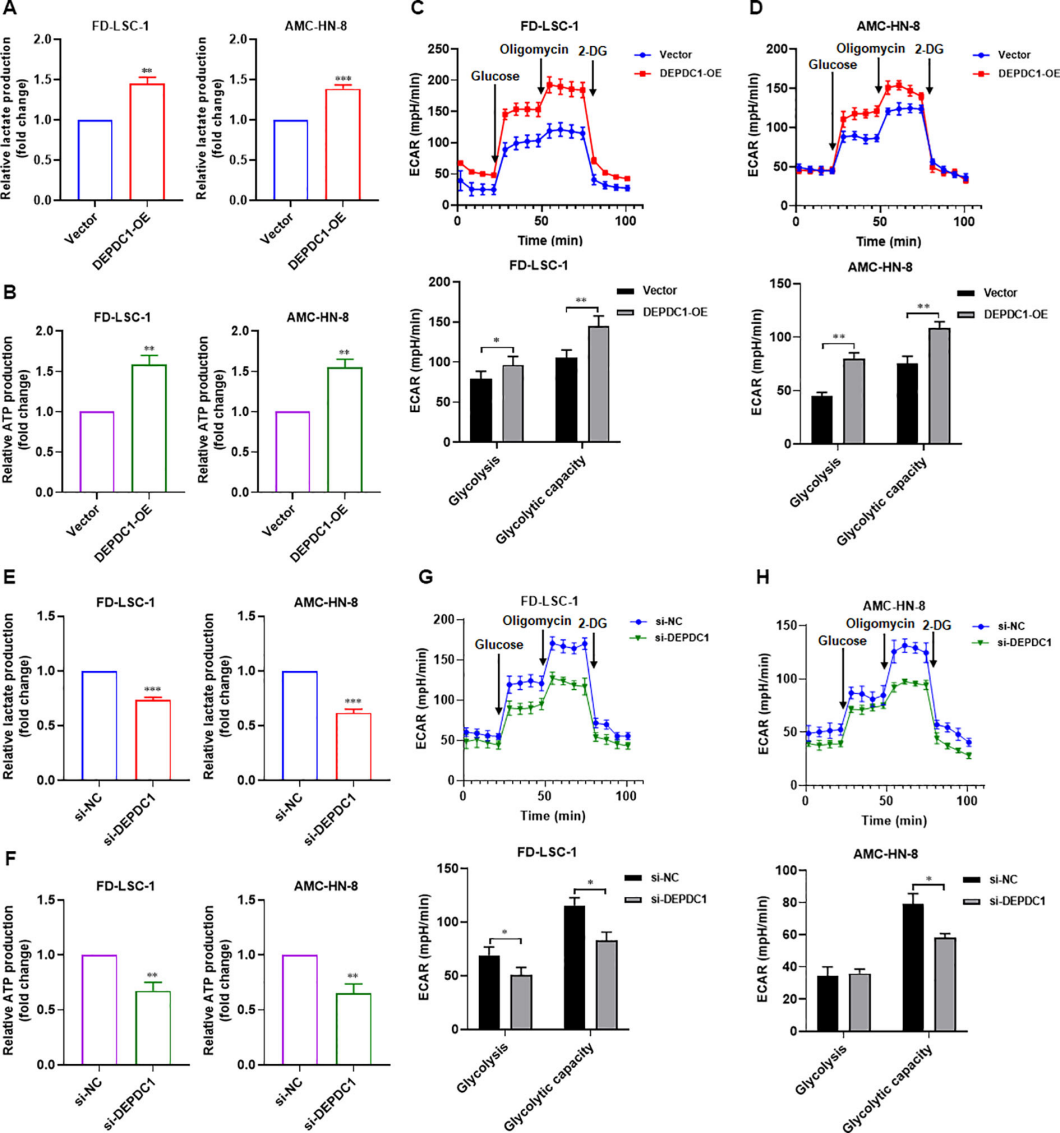
Effects of DEPDC1 on glycolysis metabolism of HNSCC cells. **(A)** Measurement of relative lactate production in FD−LSC−1 and AMC-HN-8 cells transfected with DEPDC1 overexpression plasmid (DEPDC1-OE) or control empty vector (Vector). **(B)** Measurement of relative ATP production in FD−LSC−1 and AMC-HN-8 cells transfected with DEPDC1-OE or Vector. **(C)** Measurement of ECAR in FD−LSC−1 cells transfected with DEPDC1-OE or Vector. **(D)** Measurement of ECAR in AMC-HN-8 cells transfected with DEPDC1-OE or Vector. **(E)** Measurement of relative lactate production in FD−LSC−1 and AMC-HN-8 cells transfected with si-DEPDC1 or si-NC. **(F)** Measurement of relative ATP production in FD−LSC−1 and AMC-HN-8 cells transfected with si-DEPDC1 or si-NC. **(G)** Measurement of ECAR in FD−LSC−1 cells transfected with si-DEPDC1 or si-NC. **(H)** Measurement of ECAR in AMC-HN-8 cells transfected with si-DEPDC1 or si-NC. Statistical analysis was performed using unpaired t-test for panels **(A–H)**. Data are mean ± SD of three separate experiments. *p < 0.05, **p < 0.01, ***p < 0.001.

Next, the effect of DEPDC1 on the glycolysis level of HNSCC cells was investigated. The results showed that overexpression of DEPDC1 increased glycolysis in FD-LSC-1 and AMC-HN-8 cells (**Figures 7A–D**), while knockdown of DEPDC1 decreased glycolysis of HNSCC cells (**Figures 7E–H**). To determine whether DEPDC1 drives malignant phenotypes through glycolytic upregulation, we treated DEPDC1-overexpressing HNSCC cells with the glycolysis inhibitor 2-DG. Critically, compared with the DEPDC1 overexpression alone group, 2-DG treatment reversed the promotional effect of DEPDC1 on HNSCC cell proliferation, migration, and invasion (**Supplementary Figure 3**). Collectively, these data suggest that glycolysis plays a pivotal role in DEPDC1-mediated malignant progression of HNSCC cells.

### DEPDC1 is a critical downstream effector of miR-374a-5p- and miR-374b-5p-mediated suppression of HNSCC malignant progression

**Figure 8:**
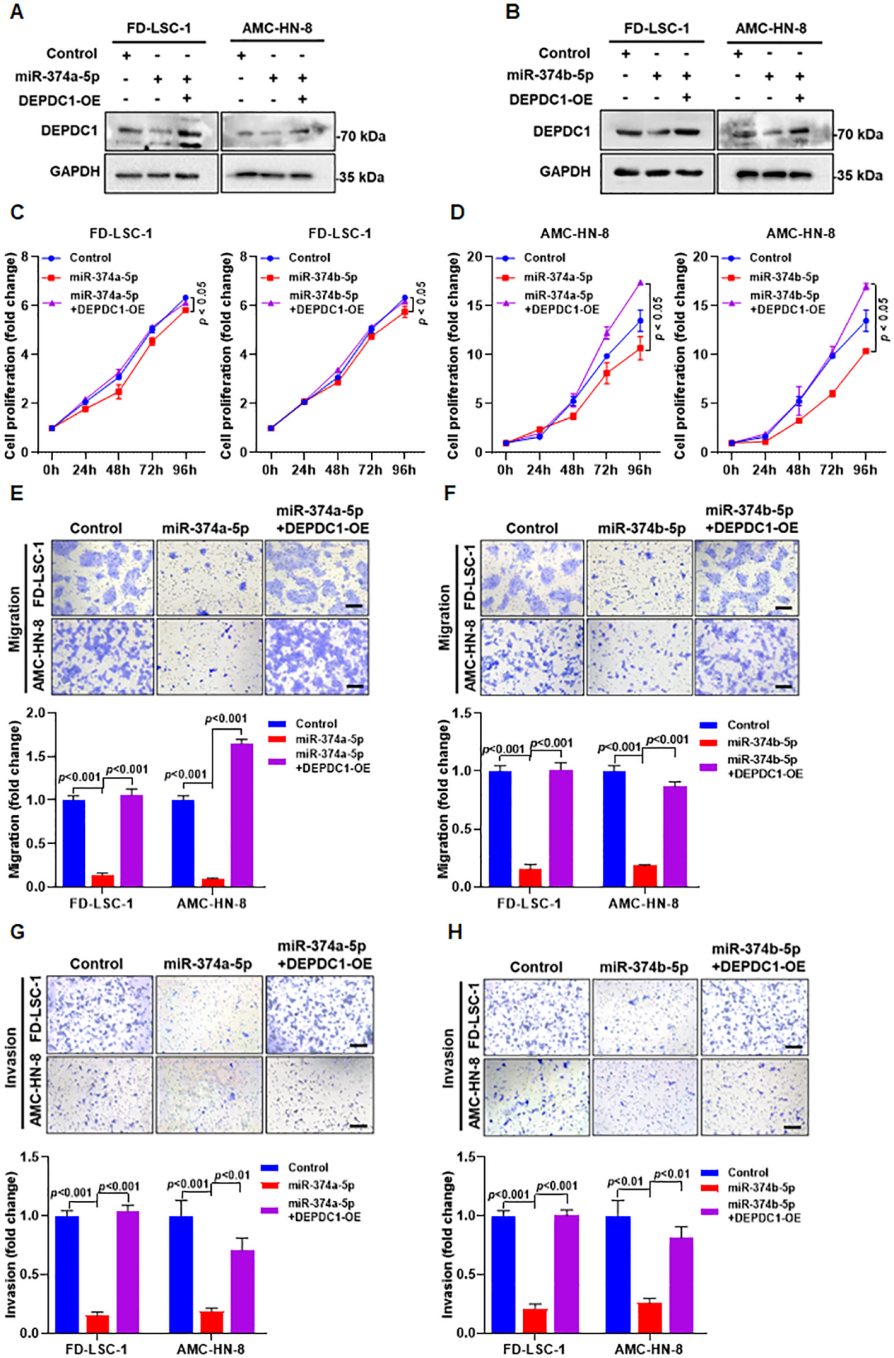
Overexpression of DEPDC1 reversed the tumor-inhibiting effects of miR-374a-5p and miR-374b-5p in HNSCC cells. **(A)** FD-LSC-1 and AMC-HN-8 cells were transfected with miR-374a-5p mimics (miR-374a-5p), or co-transfected with miR-374a-5p mimics and DEPDC1-overexpression plasmid. After 48 h of transfection, the expression of DEPDC1 was determined by western blotting. **(B)** FD-LSC-1 and AMC-HN-8 cells were transfected with miR-374b-5p mimics (miR-374b-5p), or co-transfected with miR-374b-5p mimics and DEPDC1-overexpression plasmid. After 48 h of transfection, the expression of DEPDC1 was determined by western blotting. **(C)** FD-LSC-1 cells were transfected with miR-374a-5p/miR-374b-5p mimics, or co-transfected with miR-374a-5p/miR-374b-5p mimics and DEPDC1-overexpression plasmid. Cell proliferation was measured by CCK-8 assay at the time points indicated. **(D)** AMC-HN-8 cells were transfected with miR-374a-5p/miR-374b-5p mimics, or co-transfected with miR-374a-5p/miR-374b-5p mimics and DEPDC1-overexpression plasmid. Cell proliferation was measured by CCK-8 assay at the time points indicated. **(E)** FD-LSC-1 and AMC-HN-8 cells were transfected with miR-374a-5p mimics, or co-transfected with miR-374a-5p mimics and DEPDC1-overexpression plasmid. Cell migration was measured by transwell assay. **(F)** FD-LSC-1 and AMC-HN-8 cells were transfected with miR-374b-5p mimics, or co-transfected with miR-374b-5p mimics and DEPDC1-overexpression plasmid. Cell migration was measured by transwell assay. **(G)** FD-LSC-1 and AMC-HN-8 cells were transfected with miR-374a-5p mimics, or co-transfected with miR-374a-5p mimics and DEPDC1-overexpression plasmid. Cell invasion was measured by transwell assay. **(H)** FD-LSC-1 and AMC-HN-8 cells were transfected with miR-374b-5p mimics, or co-transfected with miR-374b-5p mimics and DEPDC1-overexpression plasmid. Cell invasion was measured by transwell assay. Statistical analysis was performed using two-way ANOVA for panels **(C, D)**, and unpaired t-test for panels **(E–H)**. Scale bar, 100 μm. Data are mean ± SD of three separate experiments.

**Figure 9:**
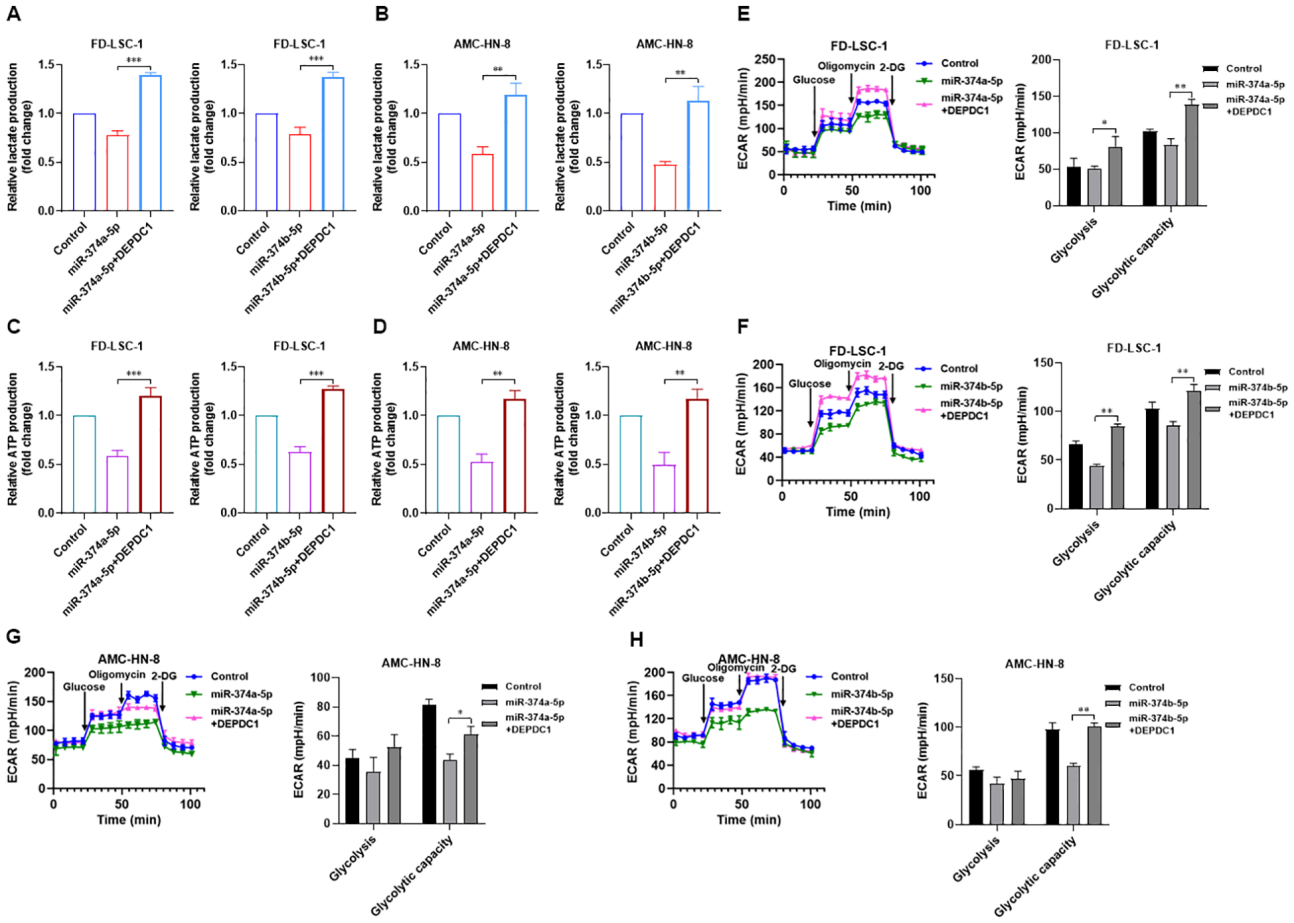
Overexpression of DEPDC1 reversed the inhibitory effects of miR-374a-5p and miR-374b-5p on glycolysis in HNSCC cells. **(A)** Measurement of relative lactate production in FD−LSC−1 cells transfected with miR-374a-5p, miR-374b-5p, co-transfected with miR-374a-5p and DEPDC1-OE, co-transfected with miR-374b-5p and DEPDC1-OE. **(B)** Measurement of relative lactate production in AMC-HN-8 cells transfected with miR-374a-5p, miR-374b-5p, co-transfected with miR-374a-5p and DEPDC1-OE, co-transfected with miR-374b-5p and DEPDC1-OE. **(C)** Measurement of relative ATP production in FD−LSC−1 cells transfected with miR-374a-5p, miR-374b-5p, co-transfected with miR-374a-5p and DEPDC1-OE, co-transfected with miR-374b-5p and DEPDC1-OE. **(D)** Measurement of relative ATP production in AMC-HN-8 cells transfected with miR-374a-5p, miR-374b-5p, co-transfected with miR-374a-5p and DEPDC1-OE, co-transfected with miR-374b-5p and DEPDC1-OE. **(E)** Measurement of ECAR in FD−LSC−1 cells transfected with miR-374a-5p, co-transfected with miR-374a-5p and DEPDC1-OE. **(F)** Measurement of ECAR in FD−LSC−1 cells transfected with miR-374b-5p, co-transfected with miR-374b-5p and DEPDC1-OE. **(G)** Measurement of ECAR in AMC-HN-8 cells transfected with miR-374a-5p, co-transfected with miR-374a-5p and DEPDC1-OE. **(H)** Measurement of ECAR in AMC-HN-8 cells transfected with miR-374b-5p, co-transfected with miR-374b-5p and DEPDC1-OE. Statistical analysis was performed using unpaired t-test for panels **(A–H)**. Data are mean ± SD of three separate experiments. *p < 0.05, **p < 0.01, ***p < 0.001.

Western blot analysis showed that, compared to transfection with miR-374a-5p mimics or miR-374b-5p mimics alone, co−transfection with either miR−374a−5p or miR−374b−5p mimics together with a DEPDC1 overexpression plasmid significantly restored DEPDC1 protein levels (**Figures 8A, B**). CCK−8 assays revealed that DEPDC1 overexpression reversed the suppressive effect of miR−374a−5p or miR−374b−5p on HNSCC cell proliferation (**Figures 8C, D**). Transwell experiments further demonstrated that DEPDC1 overexpression also abrogated the inhibitory effects of miR−374a−5p and miR−374b−5p on HNSCC cell migration and invasion (**Figures 8E–H**). Additionally, glycolysis measurements indicated that DEPDC1 overexpression could rescue the miR-374a-5p- and miR-374b-5p-induced suppression of glycolysis in both FD−LSC−1 and AMC−HN−8 cells (**Figures 9A–H**). Taken together, our results suggest that DEPDC1 is a critical downstream effector of miR-374a-5p- and miR-374b-5p-mediated suppression of HNSCC malignant progression.

### Hypermethylation of the FTX promoter suppresses the expression of miR−374a−5p and miR−374b−5p in HNSCC cells

**Figure 10:**
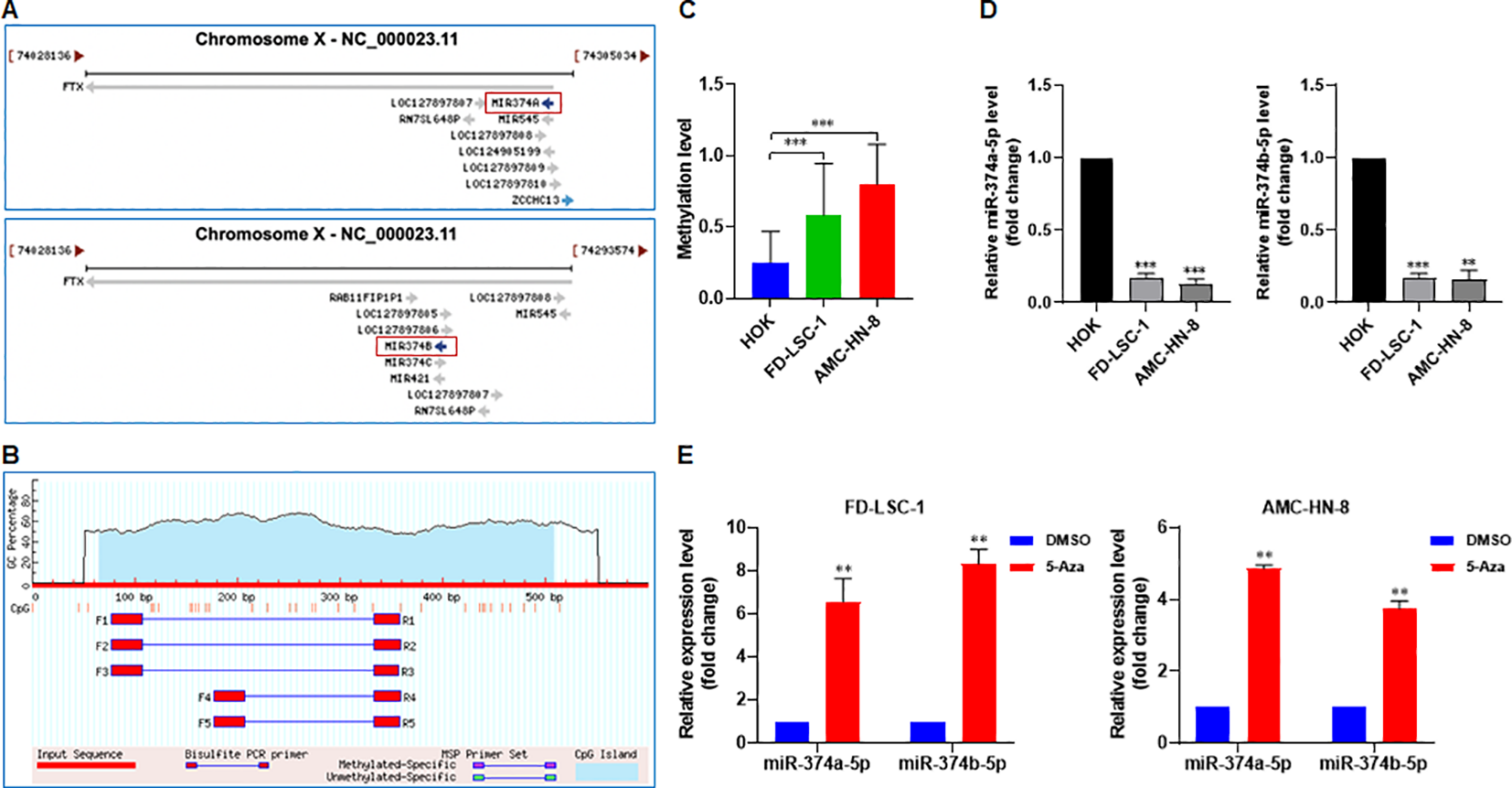
Inhibition of DNA methylation upregulates the expression of miR-374a-5p and miR-374b-5p in HNSCC cells. **(A)** Schematic representation of the genomic localization of miR−374a−5p and miR−374b−5p in the human genome. **(B)** Methylation site prediction for the parental gene FTX of miR−374a−5p and miR−374b−5p. **(C)** Promoter methylation level of the FTX gene in normal control cells HOK and HNSCC cells was analyzed by bisulfite sequencing PCR (BSP) followed by next−generation sequencing. **(D)** Expression of miR−374a−5p and miR−374b−5p in HOK, FD−LSC−1, and AMC−HN−8 cells was measured by qPCR. **(E)** FD−LSC−1 and AMC−HN−8 cells were treated with the DNA methylation inhibitor 5−Azacytidine (5−Aza) or DMSO for 24 h, after which the expression of miR−374a−5p and miR−374b−5p was assessed by qPCR. Statistical analysis was performed using unpaired t-test for panels **(C–E)**. Data are mean ± SD of three separate experiments. **p < 0.01, ***p < 0.001.

Promoter methylation is a key epigenetic mechanism that can result in gene silencing (22). Both miR-374a-5p and miR-374b-5p are transcribed from the FTX gene (**Figure 10A**). We therefore analyzed the methylation status of the FTX promoter region and identified a methylated CpG island (**Figure 10B**). Bisulfite sequencing PCR (BSP) based on next-generation sequencing revealed significantly higher methylation levels of the FTX promoter in FD-LSC-1 and AMC-HN-8 cells compared with normal HOK cells (**Figure 10C**). Consistent with this, the expression levels of both miR-374a-5p and miR-374b-5p were lower in FD-LSC-1 and AMC-HN-8 cells than in HOK cells (**Figure 10D**). These findings suggest that promoter hypermethylation may contributes to the downregulation of miR-374a-5p and miR-374b-5p in HNSCC. To further validate this, we treated HNSCC cells with the DNA methyltransferase inhibitor 5-Aza-2’-deoxycytidine (5-Aza) and measured miRNA expression by qPCR. The results demonstrated that 5-Aza treatment markedly increased the expression of both miR-374a-5p and miR-374b-5p in FD-LSC-1 and AMC-HN-8 cells (**Figure 10E**). Taken together, these data indicate that hypermethylation of the FTX promoter is a key factor contributing to the reduced expression of miR-374a-5p and miR-374b-5p in HNSCC cells.

## Discussion

Recurrence and metastasis continue to be the main clinical challenges in the treatment of HNSCC (23). Over the past decades, the survival rate of HNSCC patients has not improved substantially. Early diagnosis and intervention are key to improving therapeutic outcomes and quality of life for cancer patients (24, 25). However, effective methods for the early diagnosis of HNSCC are still lacking. As a future direction for HNSCC management, molecular diagnosis and targeted therapy urgently require the identification of biomarkers with high sensitivity and specificity.

Currently, miRNA-related studies in HNSCC cover thyroid cancer, oral squamous cell carcinoma, tongue cancer, laryngeal cancer, and oropharyngeal cancer. Recent research indicates that more than a hundred miRNAs are abnormally expressed in HNSCC, where they function as either tumor suppressors or oncogenes in a context-dependent manner (26, 27). For instance, miR−455−5p promotes migration, invasion, and EMT in oral squamous cell carcinoma (28); miR−5100 facilitates HNSCC metastasis by activating cancer−associated fibroblasts (29); and miR−503 acts as a tumor suppressor by inhibiting HNSCC cell invasion (30). Our previous work has shown that miR−145−5p and miR−1207−5p suppress the malignant progression of LSCC (31, 32). These findings highlight the scientific and translational importance of identifying functionally significant miRNAs in HNSCC and elucidating their mechanisms.

While miR−374a−5p has been implicated in inflammatory conditions such as inflammatory bowel disease and sepsis−induced acute lung injury (33, 34), its role in cancer appears tissue−specific. Liu et al. reported that miR−374a−5p inhibits papillary thyroid carcinoma growth by suppressing M2 macrophage polarization (35), whereas Son et al. found that it promotes malignancy in triple−negative breast cancer (36). Similarly, miR−374b−5p suppresses proliferation, migration, and EMT in ovarian cancer (37) but enhances proliferation and migration while inhibiting apoptosis in glioblastoma (38). These seemingly contradictory results suggest that the functions of miR−374a−5p and miR−374b−5p are highly context−dependent, underscoring the need to clarify their roles in HNSCC.

In this study, we found that both miR−374a−5p and miR−374b−5p are significantly downregulated in HNSCC tissues, pointing to their potential regulatory importance. Functional assays demonstrated that overexpression of either miRNA inhibits proliferation, invasion, migration, and glycolysis in HNSCC cells. *In vivo* experiments further confirmed that both miRNAs suppress tumorigenesis of HNSCC cells. Together, these results support a tumor−suppressive role for miR−374a−5p and miR−374b−5p in HNSCC.

Both miR-374a-5p and miR-374b-5p share an identical seed sequence, and these two miRNAs have highly overlapping target genes. Bioinformatics analysis suggested that the target genes of miR-374a-5p and miR-374b-5p are mainly implicated in regulating extracellular matrix organization, DNA replication, cell cycle, cell division, chromatin separation, PI3K/Akt, TGF-β, and other biological processes and signaling pathways. Combining literature reports and our previous research data, we identified an important candidate downstream target gene, DEPDC1. The DEPDC1 gene encodes a highly conserved protein that is involved in cell proliferation, cell cycle, and signal transduction processes (39). Studies have shown that DEPDC1 functions as an oncogene in gastric cancer, osteosarcoma, hepatocellular carcinoma, renal cell carcinoma, and other malignant tumors, promoting the malignant progression of cancer cells (40–43). By analyzing TCGA and GEO RNA-seq data, we found that DEPDC1 is highly expressed in HNSCC, and its high expression is associated with poor prognosis in HNSCC patients, suggesting that DEPDC1 might be an oncogene in HNSCC. We further investigated the effects of DEPDC1 on the proliferation, migration, and invasion of HNSCC cells through gain-and-loss of function experiments. The results demonstrated that overexpression of DEPDC1 promotes the proliferation, migration, and invasion of HNSCC cells, while knockdown of DEPDC1 inhibits these processes. Our results also demonstrated that DEPDC1 enhances glycolysis in HNSCC cells. These results suggest that DEPDC1 plays an opposite role in HNSCC compared with miR-374a-5p and miR-374b-5p. Interestingly, the rescue experiments revealed that overexpression of DEPDC1 could reverse the inhibitory effect of miR-374a-5p and miR-374b-5p on the proliferation, migration, and invasion abilities of HNSCC cells, indicating that miR-374a-5p and miR-374b-5p mainly inhibit the malignant phenotype of HNSCC cells by downregulating the expression level of DEPDC1. Furthermore, our results revealed that hypermethylation of the promoter region is an important upstream regulatory factor that results in downregulation of miR-374a-5p and miR-374b-5p in HNSCC cells. Notably, the present study was primarily conducted in cell lines and xenograft models. Prospective validation in HNSCC patient cohorts or patient-derived xenograft models is therefore essential to confirm the clinical relevance of miR-374a-5p and miR-374b-5p as therapeutic targets.

In conclusion, the findings of this study indicate that miR−374a−5p and miR−374b−5p, which are downregulated in HNSCC, act as tumor suppressors by inhibiting cell proliferation, invasion, migration, glycolytic activity, *in vivo* tumorigenicity, and the EMT process in HNSCC cells. Mechanistic investigations reveal that these miRNAs exert their tumor−suppressive functions by downregulating the expression of the target gene DEPDC1. Moreover, our findings demonstrate that promoter hypermethylation plays a key role in suppressing miR−374a−5p and miR−374b−5p expression in HNSCC cells. Collectively, these results provide new insights into the molecular mechanisms underlying HNSCC progression and highlight potential biomarkers and therapeutic targets for the diagnosis and treatment of HNSCC.

## Data Availability

The datasets presented in this study can be found in online repositories. The names of the repository/repositories and accession number(s) can be found in the article/**Supplementary Material**.

## Supplementary material

Supplementary Figure 1 Inhibition of miR-374a-5p and miR-374b-5p promotes proliferation, migration, and invasion in HNSCC cells. **(A)** Proliferation of FD−LSC−1 cells transfected with miR−374a−5p inhibitor, miR−374b−5p inhibitor, or NC inhibitor was assessed by CCK−8 assay at the indicated time points. **(B)** Proliferation of AMC-HN-8 cells transfected with miR−374a−5p inhibitor, miR−374b−5p inhibitor, or NC inhibitor was assessed by CCK−8 assay at the indicated time points. **(C)** Migration ability of FD−LSC−1 and AMC−HN−8 cells transfected with miR−374a−5p inhibitor, miR−374b−5p inhibitor, or NC inhibitor was evaluated by transwell assay. **(D)** Invasion ability of FD−LSC−1 and AMC−HN−8 cells transfected with miR−374a−5p inhibitor, miR−374b−5p inhibitor, or NC inhibitor was evaluated by transwell assay. Scale bar, 100 μm. Statistical analysis was performed using two-way ANOVA for panels **(A, B)**, and unpaired t-test for panels **(C, D)**. Data are mean ± SD of three separate experiments. **p* < 0.05, ***p* < 0.01, ****p* < 0.001.

Supplementary Figure 2 Validation of the knockdown efficiency of DEPDC1−targeting siRNAs in HNSCC cells. FD−LSC−1 and AMC−HN−8 cells were transfected with DEPDC1−targeting siRNAs (si−DEPDC1−1, si−DEPDC1−2) or negative control siRNA (si−NC). After 48 h, DEPDC1 expression was measured by qPCR. Statistical analysis was performed using unpaired t-test. Data are mean ± SD of three separate experiments. ****p* < 0.001.

Supplementary Figure 3 Inhibition of glycolysis reverses the DEPDC1−mediated promotion of proliferation, migration, and invasion in HNSCC cells. FD−LSC−1 and AMC−HN−8 cells were transfected with a DEPDC1 overexpression plasmid (DEPDC1−OE), then treated with glycolysis inhibitor 2-DG. **(A)** Cell proliferation was assessed by CCK−8 assay. **(B)** Cell migration was evaluated by transwell assay. **(C)** Cell invasion was evaluated by transwell assay. Statistical analysis was performed using two-way ANOVA for panel **(A)**, and unpaired t-test for panels **(B, C)**. Scale bar, 100 μm. Data are mean ± SD of three separate experiments. ***p* < 0.01, ****p* < 0.001.
