# Supplementary figures and images for: Methylation-regulated miR-374a-5p and miR-374b-5p suppress glycolysis and malignant progression of head and neck squamous cell carcinoma by targeting DEPDC1
Source: Front Oncol. 2026 May 15;16:1816226. doi: 10.3389/fonc.2026.1816226 (PMC13218900; doi:10.3389/fonc.2026.1816226)

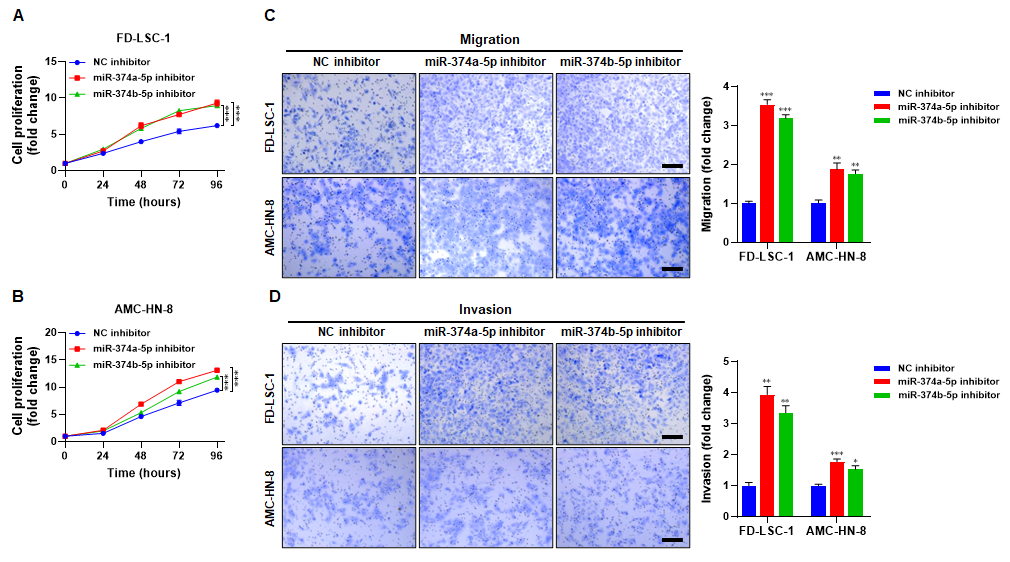

Supplement: Supplementary Figure 1 — Inhibition of miR-374a-5p and miR-374b-5p promotes proliferation, migration, and invasion in HNSCC cells. (A) Proliferation of FD−LSC−1 cells transfected with miR−374a−5p inhibitor, miR−374b−5p inhibitor, or NC inhibitor was assessed by CCK−8 assay at the indicated time points. (B) Proliferation of AMC-HN-8 cells transfected with miR−374a−5p inhibitor, miR−374b−5p inhibitor, or NC inhibitor was assessed by CCK−8 assay at the indicated time points. (C) Migration ability of FD−LSC−1 and AMC−HN−8 cells transfected with miR−374a−5p inhibitor, miR−374b−5p inhibitor, or NC inhibitor was evaluated by transwell assay. (D) Invasion ability of FD−LSC−1 and AMC−HN−8 cells transfected with miR−374a−5p inhibitor, miR−374b−5p inhibitor, or NC inhibitor was evaluated by transwell assay. Scale bar, 100 μm. Statistical analysis was performed using two-way ANOVA for panels (A, B), and unpaired t-test for panels (C, D). Data are mean ± SD of three separate experiments. *p < 0.05, **p < 0.01, ***p < 0.001. [file Image1.tif]

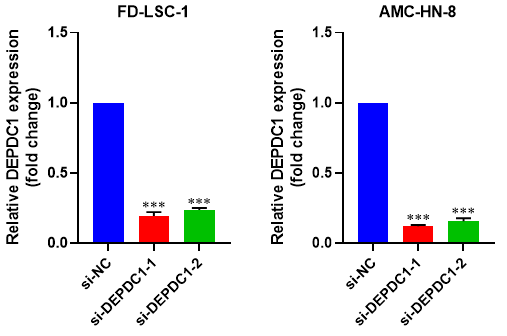

Supplement: Supplementary Figure 2 — Validation of the knockdown efficiency of DEPDC1−targeting siRNAs in HNSCC cells. FD−LSC−1 and AMC−HN−8 cells were transfected with DEPDC1−targeting siRNAs (si−DEPDC1−1, si−DEPDC1−2) or negative control siRNA (si−NC). After 48 h, DEPDC1 expression was measured by qPCR. Statistical analysis was performed using unpaired t-test. Data are mean ± SD of three separate experiments. ***p < 0.001. [file Image2.tif]

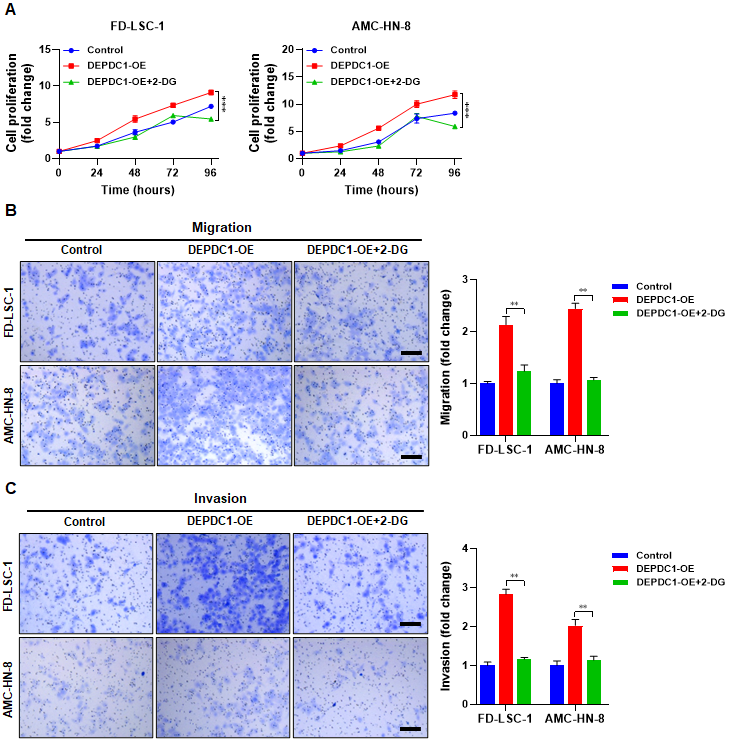

Supplement: Supplementary Figure 3 — Inhibition of glycolysis reverses the DEPDC1−mediated promotion of proliferation, migration, and invasion in HNSCC cells. FD−LSC−1 and AMC−HN−8 cells were transfected with a DEPDC1 overexpression plasmid (DEPDC1−OE), then treated with glycolysis inhibitor 2-DG. (A) Cell proliferation was assessed by CCK−8 assay. (B) Cell migration was evaluated by transwell assay. (C) Cell invasion was evaluated by transwell assay. Statistical analysis was performed using two-way ANOVA for panel (A), and unpaired t-test for panels (B, C). Scale bar, 100 μm. Data are mean ± SD of three separate experiments. **p < 0.01, ***p < 0.001. [file Image3.tif]
